# Phosphorylation of unique domains of Src family kinases

**DOI:** 10.3389/fgene.2014.00181

**Published:** 2014-06-30

**Authors:** Irene Amata, Mariano Maffei, Miquel Pons

**Affiliations:** Biomolecular NMR Laboratory, Department of Organic Chemistry, University of BarcelonaBarcelona, Spain

**Keywords:** IDPs, IDRs, phosphorylation, unique domain, SFKs, Src

## Abstract

Members of the Src family of kinases (SFKs) are non-receptor tyrosine kinases involved in numerous signal transduction pathways. The catalytic, SH3 and SH2 domains are attached to the membrane-anchoring SH4 domain through the intrinsically disordered “Unique” domains, which exhibit strong sequence divergence among SFK members. In the last decade, structural and biochemical studies have begun to uncover the crucial role of the Unique domain in the regulation of SFK activity. This mini-review discusses what is known about the phosphorylation events taking place on the SFK Unique domains, and their biological relevance. The modulation by phosphorylation of biologically relevant inter- and intra- molecular interactions of Src, as well as the existence of complex phosphorylation/dephosphorylation patterns observed for the Unique domain of Src, reinforces the important functional role of the Unique domain in the regulation mechanisms of the Src kinases and, in a wider context, of intrinsically disordered regions in cellular processes.

## INTRODUCTION

**FIGURE 1:**
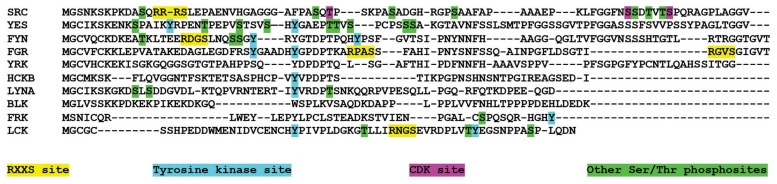
**Phosphorylation and lipid binding sites in the Unique domain of SFK.** Alignment of the UD sequences of the ten SFK. The alignment has been manually edited to emphasize the similarities in regions that are known to be functionally relevant in at least one SFK member. Residues highlighted in green are identified as phosphorylation sites in PhosphoSitePlus (Hornbeck et al., 2012). Conservation of phosphorylation sites was used as a reference in the alignment presented.

The Src kinase family is composed of 10 proteins: Src, Frk, Lck, Lyn, Blk, Hck, Fyn, Yrk, Fgr, and Yes. Src family kinases (SFKs) are membrane-associated, non-receptor tyrosine kinases that act as important signaling intermediaries regulating a variety of outputs, such as cell proliferation, differentiation, apoptosis, migration, and metabolism. All SFKs share the same domain arrangement: a large catalytic C-terminal domain is preceded by regulatory Src 2 and 3 homology domains (SH2 and SH3, respectively) and the membrane-anchoring SH4 N-terminal region, which contain myristoylation and palmitoylation sites as well as positively charged residues. The SH3 and SH4 domains are linked by an intrinsically disordered segment of 50–90 residues, called the Unique domain (UD) because of the lack of sequence similarity among the different SFKs. However, the UD of each individual SFK member is well conserved between different organisms suggesting a more specific role than that of a simple spacer. Figure 1 shows the sequences of the UDs of the 10 SFKs, highlighting known phosphorylation sites.

Efforts to find specific UD functions during the last two decades have confirmed an active role of the UD in the regulation of SFK members. In the case of Lck, the UD mediates association with the cytoplasmic tail of CD4 and CD8α through a zinc clasp structure (Kim et al., 2003). For most of the other SFK members the detailed mechanisms remain obscure. The Unique domains of Fyn and Lyn were observed to be cleaved during induction of apoptosis in intact hematopoietic cells, revealing a novel mechanism for the specific regulation of different SFKs, with important consequences for their cellular localization and activity (Luciano et al., 2001). Several SFKs contain residues in the N-terminal region that are phosphorylated and dephosphorylated in cellular processes (Joung et al., 1995; Hansen et al., 1997; Johnson et al., 2000). Moreover, swapping the Unique domains of Src and Yes interchanges the functional specificity of the two SFKs (Hoey et al., 2000; Summy et al., 2003; Werdich and Penn, 2005). The versatility and relevance of the active role of the Unique domain in Src function was confirmed by the discovery of binding by the Unique domain to different targets, such as acidic lipids, the SH3 domain, and calmodulin (Pérez et al., 2013). The amino-acid sequence of the UD region responsible for some of these interactions is conserved in Src, Fyn, and Yes. The three proteins are co-expressed in many tissues and are partially redundant, in the sense that deficiency in one of them can be compensated by the action of the other two.

The involvement of SFK signaling in growth, motility and cell survival makes them important oncology targets. Activation of SFKs is tightly regulated in healthy cells (Boggon and Eck, 2004), while the kinase activation is often deregulated in cancer, giving rise to altered cellular shape, function, and growth (Vlahovic and Crawford, 2003). Among the SFKs, Src is the most studied and the most commonly discussed in the context of cancer (Yeatman, 2004). However, there has been growing interest in the other SFKs in both normal physiological and pathological states.

The UD is an intrinsically disordered region (IDR). Intrinsically disordered proteins (IDPs) are relatively more prevalent among signaling and cancer-related proteins (Iakoucheva et al., 2002). The connection between disorder and diseases such as cancer, neurodegenerative conditions, amyloidosis, cardiovascular disease, and diabetes has been extensively explored in recent reviews (Uversky et al., 2008; Uros et al., 2009). Diseases arising from structural changes in proteins, loosely grouped as “conformational diseases” are caused not only by protein misfolding, but also by failures in post-translational modifications that result in aberrant interactions with physiological partners (Uversky et al., 2008). Remarkably, mutations in disordered regions can result in the loss of important post-translational modification sites, leading to disease (Li et al., 2010). The recognition of the crucial role of IDPs in a number of diseases has heralded a new era in the design of drugs (Chen et al., 2006).

While the catalytic and Src homology domains of SFKs have long been subjects of investigation, the study of the regulatory mechanisms involving the intrinsically disordered UD has only recently started gaining momentum. Phosphorylation events are often associated with the regulation of important functional regions. In this mini-review we discuss the current knowledge about the phosphorylation events taking place in the Unique domains of SFKs.

## PHOSPHORYLATION OF THE UNIQUE DOMAIN OF Lck

The Unique domain linking the SH4 and the SH3 domain of Lck is one of the smallest of the family, with only 60 amino acids. During T-cell activation, Ser59 in the Unique N-terminal region of Lck is phosphorylated (Watts et al., 1993; Winkler et al., 1993). The Unique domain of Lck contains a proline-rich region surrounding Ser59 (^56^PPASP^60^). Joung et al. (1995) found that modifications of Ser59 in the Unique N-terminal domain of tyrosine kinase Lck regulate specificity of its SH2 domain. Later on, Ser59 was identified as a site of *in vivo* mitotic phosphorylation in Lck (Kesavan et al., 2002). More recently, a new target was found for Lck: the protein Nck (Vázquez, 2007), consisting of one SH2 domain and three SH3 domains, which is known to link receptor tyrosine kinases to downstream proteins, and to be active in actin polymerization. It was also reported that Nck binds in T cells to the CD3 subunit of the T-cell antigen receptor (TCR) following TCR engagement. Interestingly, the inter-molecular interaction between Lck and Nck was observed to be disrupted by phosphorylation at Ser59 within the Unique domain of Lck. Wild-type Lck (wt-Lck) and a Ser59Asp-Lck mutant transfected into Lck-deficient JCaM1.6 cells showed differences in the activation of proximal versus distal signaling events (Vázquez, 2007).

## PHOSPHORYLATION OF THE UNIQUE DOMAIN OF Hck

Hematopoietic cell kinase (Hck) is a potential drug target for cancer and HIV infections. High levels of Hck are associated with drug resistance in chronic myeloid leukemia and Hck activity has been connected with HIV-1 (Tintori et al., 2013). An important insight into the activation mechanism of this SFK member was the discovery that Hck is capable of performing autophosphorylation in its UD. Autophosphorylation of the activation loop in the kinase domain is a common process for SFK activation. Autophosphorylation of recombinant Hck leads to a 20-fold increase in its specific enzymatic activity (Johnson et al., 2000). Hck was found to autophosphorylate readily to a stoichiometry of 1.3 mol of phosphate per mol of enzyme, indicating that the kinase autophosphorylated at more than one site. In particular, Johnson and collaborators discovered –- *in vitro* as well as *in vivo*–-that Hck can undergo autophosphorylation at two different sites: Tyr388, which is located at the consensus autophosphorylation site in the well-characterized activation loop of similar kinases, and Tyr29, which is located in its intrinsically disordered Unique domain. By inspecting the activities and levels of phosphorylation of recombinant Hck mutants containing either the point mutation Tyr388Phe or Tyr29Phe, they demonstrated that phosphorylation at Tyr29 makes a crucial contribution to the activation of Hck through its autophosphorylation. Regulation of the catalytic activity by phosphorylation at Tyr29 in the Unique domain of Hck suggests that autophosphorylation within the N-terminal Unique region may also be an additional mechanism of regulation of other Src family tyrosine kinases.

## TYROSINE PHOSPHORYLATION IN OTHER UNIQUE DOMAINS: Lyn, Yes, Fgr, AND Frk

Interestingly, the tyrosine residue in position 32 of Lyn is located in an 8-residue-long sequence common to Lyn and Hck. EGFR phosphorylates the p56 isoform of Lyn, p56^Lyn^, at Tyr32, which then phosphorylates MCM7, a licensing factor critical for DNA replication. Phosphorylation at Tyr600 of MCM7 increases its association with other minichromosome maintenance complex proteins, thereby promoting DNA synthesis complex assembly and cell proliferation. Both p56^Lyn^ Tyr32 and MCM7 Tyr600 phosphorylation are enhanced in proliferating cells and correlated with poor survival of breast cancer patients (Huang et al., 2013).

All Unique domains contain aromatic residues, a rare feature in intrinsically disordered domains. A tyrosine residue is present between positions 25 and 34 in seven SFKs, and is known to be phosphorylated in five of them: Lyn, Hck, Lck (Hornbeck et al., 2012), Yes (Ariki et al., 1997), and Frg (Oppermann et al., 2009). Phosphorylation of Tyr34 of Fgr has been observed my mass spectrometry mainly in samples from leukemia patients.

Frk is the only SFK that is not myristoylated. Although it contains three tyrosine residues in its Unique domain, phosphorylation has only been reported for Tyr46, at the interface between the Unique and SH3 domains. The modification was observed by mass spectrometry mainly in samples from lung, liver, and gastric cancer (Stokes et al., 2012).

## PHOSPHORYLATION OF THE UNIQUE DOMAIN OF Fyn

Fyn is ubiquitously expressed together with Src and Yes, whereas other members of the Src family are expressed only in specific cell types. Fyn is primarily localized to the cytoplasmic leaflet of the plasma membrane, where it phosphorylates tyrosine residues on key targets involved in a variety of different signaling pathways. Fyn characterization has been mainly focused on its immune and neurological function. However, Fyn has also been recognized as an important mediator of mitogenic signaling and regulator of cell-cycle entry, growth, and proliferation, integrin-mediated interactions as well as cell adhesion and migration (Kawakami et al., 1988; Chen et al., 2001; Li et al., 2003; Kim et al., 2010). In particular, Fyn is over-expressed in several cancers, such as glioblastoma multiformae and melanoma. The role of Fyn over-expression in these systems, however, has not been well defined as yet.

Fyn presents multiple phosphorylation sites that can affect its kinase activity. Activation of Platelet-Derived Growth Factor (PDGF) β-receptor by binding to PDGF leads to activation of a member of the SFK (Alonso et al., 1995). The Unique domain of Fyn was found to be phosphorylated at Tyr28 (Hansen et al., 1997). The functional role of this phosphorylation was confirmed by the observation of significantly reduced activation following PDGF stimulation of a Fyn mutant in which Tyr28 was replaced by phenylalanine (Hansen et al., 1997). It was also proposed that the autophosphorylation of the N-terminal tyrosine residues plays a key role in the activation of Fyn, by complementing the PDGF receptor-induced phosphorylation of Tyr28.

Serine 21 within the UD of Fyn is part of a RxxS motif targeted by protein kinase A (PKA). Mutation of Ser21 to Alanine (Ser21Ala-Fyn) blocks PKA phosphorylation of Fyn and alters its tyrosine kinase activity (Yeo et al., 2011). In the same work, the authors showed that the over-expression of Ser21Ala-Fyn mutant in cells lacking Src/Yes/Fyn kinases (SYF cells) led to decreased tyrosine phosphorylation of focal adhesion kinase, resulting in reduced focal adhesion targeting, and slow lamellipodia dynamics and cell migration. These important changes in cell motility demonstrate a key role of UD phosphorylation at Ser21 in the Fyn kinase activity that controls assembly and disassembly of focal adhesions in response to signals arising from cell-extracellular matrix interactions.

Protein kinase A is a crucial component of integrin-mediated signaling pathways. Interaction of cells with their substrate or adhesion to other cells leads to activation of PKA (Whittard and Akiyama, 2001), and induce the phosphorylation of a variety of protein substrates (Walsh and Van Patten, 1994). Four SFKs (Fyn, Src, Lck, and Fgr) contain a conserved RxxS motif suggesting they can be regulated by PKA.

## PHOSPHORYLATION OF THE UNIQUE DOMAIN OF Src

Src is a non-receptor protein tyrosine kinase with a key role in regulating cell-to-matrix adhesion, migration, and junctional stability (Frame, 2004). Thus, precise regulation of Src activity is critical for normal cell growth. The inactive state of Src is obtained by phosphorylated tyrosine near the C-terminus of Src (Tyr530 in mammalian Src; Tyr527 in chicken Src), which is recognized by its SH2 domain, while the SH3 domain interacts with a poly-proline motif located in the linker region between the SH2 and kinase domains; these intramolecular interactions restrict access to the kinase domain (Xu et al., 1997). Dephosphorylation of Tyr530 is followed by autophosphorylation at Tyr419, leading to full activation of the kinase. Active Src may be deactivated by rephosphorylation of Tyr530 by C-terminal Src kinase (Csk; Piwnica-Worms et al., 1987; Nada et al., 1991; Brown, 1996).

Although the phosphorylation of Src at Ser17 by PKA (cAMP-dependent protein kinase) is a well-characterized process, its biological significance remains unclear (Obara et al., 2004). Potential roles in protein–protein interactions or cellular localization have been postulated for this phosphorylation site. For instance, it has been observed that the treatment of 3T3 fibroblasts with PDGF results in the translocation of Src from the plasma membrane to the cytosol, concomitant with an increase in phosphorylation of Ser17 by PKA (Walker et al., 1993). This observation suggests that this phosphorylation could interfere with the electrostatic interactions that act to anchor Src to the lipid bilayer. PKA phosphorylation of Src at Ser17 is also required in cAMP activation of Rap1, inhibition of extracellular signal-regulated kinases, and inhibition of cell growth, although the mechanism by which this phosphorylation mediates these processes is not known (Obara et al., 2004).

**FIGURE 2:**
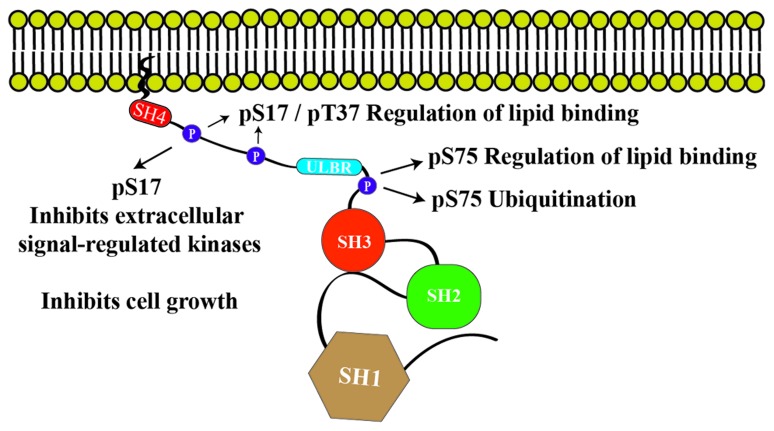
**Phosphorylation sites in the UD of Src and their relationship with the two lipid-binding regions: the classical SH4 domain and the ULBR**.

Interaction of Src with lipids is not restricted to the SH4 domain (Figure 2). The unique lipid binding region (ULBR) was discovered following NMR observations that revealed a partially structured region within the UD of Src (Pérez et al., 2009). While phosphorylation of Ser17 by PKA disturbed the interaction of the SH4 domain with lipids, phosphorylation of Thr37 and Ser75 by p25-Cdk5 decreased lipid binding by the ULBR (Pérez et al., 2009). Cross-effects were observed, suggesting a cooperative interaction of the two lipid binding regions with membranes. Conformational effects of phosphorylation at Ser17, Thr37, and Ser75 in the isolated UD are strictly local, indicating that electrostatic repulsion of the phosphorylated residues with the negatively charged lipids is the main mechanism by which lipid binding is disrupted.

The ULBR major regulatory role of the UD was shown experimentally in *Xenopus laevis* oocytes. Mutations abolishing lipid binding by the ULBR but not affecting the SH4 domain resulted in a conditional lethal phenotype after progesterone induced maturation (Pérez et al., 2013).

Previous results had identified a functional role for Thr37 and Ser75 (Thr34 and Ser72 in chicken Src) as well as Thr46 in chicken Src (with no correspondence in humans). These residues are phosphorylated by cyclin-dependent kinase 1 (Cdk1/Cdc2) during mitosis. These phosphorylations were found to activate Src by disrupting the interaction between the SH2 domain and Tyr527/Tyr530 (chicken/human) and facilitating the dephosphorylation by protein tyrosine phosphatases (Shenoy et al., 1992; Stover et al., 1994).

In addition (or possibly related to) its effect on lipid binding, phosphorylation of Ser75 is important in other aspects of Src regulation. Mitosis-independent phosphorylation of this site was observed in neurons and in certain cancer cell lines; this phosphorylation was shown to be due to Cdk5, a widely distributed proline-directed kinase with a substrate specificity similar to Cdk1 (Kato and Maeda, 1999). Active Src is reported to be irreversibly destroyed by Cullin-5-dependent ubiquitination and proteosomal degradation (Hakak and Martin, 1999; Laszlo and Cooper, 2009). More recently, it was shown that phosphorylation at Ser75 promotes the ubiquitin-dependent degradation of Src (Pan et al., 2011). Ser75-Src phosphorylation in epithelial cells was found to depend on the activation state of Src: only active Src was phosphorylated and eventually marked for ubiquitination. Thus, Cdk5-dependent phosphorylation of Ser75 within the UD of Src represents a mechanism to restrict the availability of active Src (Pan et al., 2011). Sequence alignment suggests that serine residues are present in homologous positions of Yes and possibly Frk.

NMR and mass-spectrometry studies of human Src UD added to *X. laevis* egg extracts showed the phosphorylation of Ser75 and Ser69, as well as Ser 17 (Pérez et al., 2013). Phosphorylation of Ser75 and Ser69 seem to be mutually interfering *in vitro*. Interestingly, *X. laevis* Src has a glycine residue at position 69 and phosphorylation of Ser69 in human Src had only been previously detected by mass spectrometry in extracts of cancer lines HCT116 and MDA-MB-435S (Oppermann et al., 2009). It is tempting to speculate that pathological phosphorylation of Ser 69 in human cells could result in decreased phosphorylation of Ser75 and reduced degradation of active Src, leading to an oncogenic phenotype caused by Src overactivation.

The interplay between various phosphorylation sites within the UD emphasizes its role as a signaling integration hub. Further input signals, in addition to phosphorylation, include calcium-dependent interaction with calmodulin and the allosteric interaction with Src SH3 domain (Pérez et al., 2013; Figure 2). A further level of cross-talk between various phosphorylation events was observed when the time-dependent phosphorylation of the UD was studied by real-time NMR in cell extracts (Amata et al., 2013). In these experiments it was observed that the activity of a PKA-like kinase, which phosphorylates Ser17, also repressed the phosphatase(s) that catalyzed the dephosphorylation of Ser75. Thus, inhibition of PKA activity resulted in dephosphorylation of both sites. Similarly, inhibition of Cdk activity resulted in a reduction in the steady-state phosphorylation of both sites. On the other hand, addition of PKA caused a robust phosphorylation of Ser75 by preventing the action of the phosphatases that reverse the effect of Cdks. These results show that the phosphorylation state of the UD of Src represents a sensor of the kinase-phosphatase network active at any given moment in the cell. Experimental access to this information is possible by using real-time NMR techniques (Selenko et al., 2008; Theillet et al., 2012; Thongwichian and Selenko, 2012; Amata et al., 2013). In contrast to folded domains, intrinsically disordered domains, like the UD of Src, provide *in vivo* NMR resolution comparable to that obtainable *in vitro* (Kosol et al., 2013).

## CONCLUDING REMARKS

The study of the UDs of SFKs, particularly that of Src, provides an example of multilevel regulation through phosphorylation of a membrane-bound intrinsically disordered domain. The occurrence of membrane tethering among IDRs is very common, although its relevance is not always recognized. The results arising from studies of UDs should stimulate further research to uncover similar mechanisms beyond SFKs. In particular, switchable internal lipid binding sites (the ULBR in the case of Src) in disordered domains anchored to membranes by a second, more stable, binding site close to the protein termini (the SH4 domain in the case of SFKs) can modulate the position of the active domains (the kinase or regulatory domains in the case of SFKs) with respect to the membrane surface. Lipid binding by the internal site can switch off the access of the active domains to target sites of membrane-anchored substrates/partners located beyond a particular distance from the membrane surface or, conversely, facilitate the interaction with sites near the membrane surface. Phosphorylation or other modifications preventing the interaction of the internal site would result in a release of the active domains, which can then reach sites located further apart from the membrane surface or disfavor the interaction with membrane-proximal sites. This represents a new compartmentalization mechanism based on the relative position of interacting sites with respect to the membrane surface. This mechanism enables modulating the functional interactions between two proteins anchored next to each other in the membrane.

## Conflict of Interest Statement

The authors declare that the research was conducted in the absence of any commercial or financial relationships that could be construed as a potential conflict of interest.
